# Supplementary material for: Selection of housekeeping genes as internal controls for quantitative RT-PCR analysis of the veined rapa whelk (Rapana venosa)
Source: PeerJ. 2017 May 31;5:e3398. doi: 10.7717/peerj.3398 (PMC5455708; doi:10.7717/peerj.3398)
Supplement: Figure S2 [file peerj-05-3398-s003.pdf]

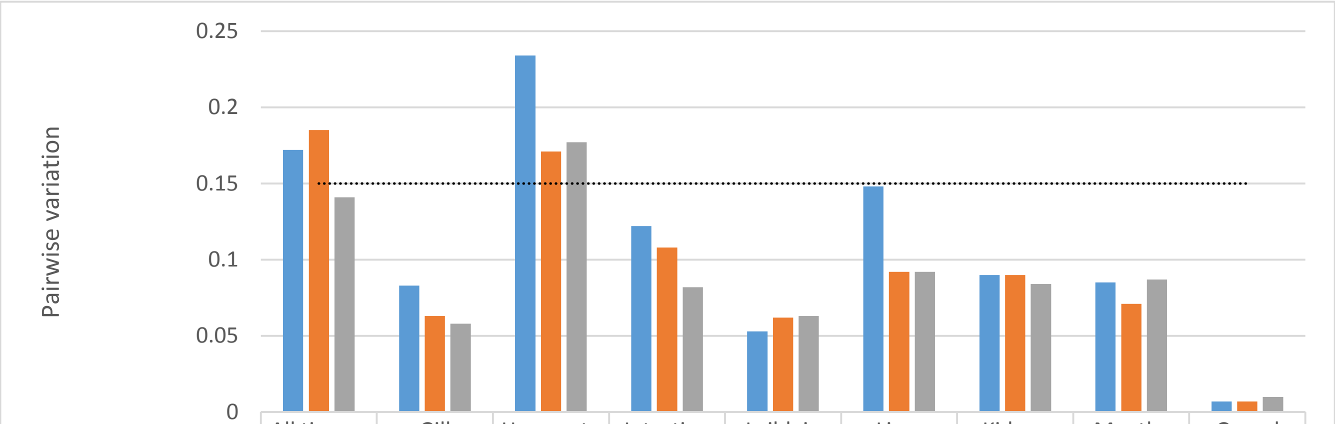

	All tissues	Gill	Hemocyte	Intestine	Leiblein	Liver	Kidney	Mantle	Gonad
V2/3	0.172	0.083	0.234	0.122	0.053	0.148	0.09	0.085	0.007
V3/4	0.185	0.063	0.171	0.108	0.062	0.092	0.09	0.071	0.007
V4/5	0.141	0.058	0.177	0.082	0.063	0.092	0.084	0.087	0.01
cut-off value	0.15	0.15	0.15	0.15	0.15	0.15	0.15	0.15	0.15

Figure S2. The number of reference genes required for accurate normalization of eight different tissues based on pairwise variation between candidate genes by GeNorm.
