# Supplementary material for: Selection of housekeeping genes as internal controls for quantitative RT-PCR analysis of the veined rapa whelk (Rapana venosa)
Source: PeerJ. 2017 May 31;5:e3398. doi: 10.7717/peerj.3398 (PMC5455708; doi:10.7717/peerj.3398)
Supplement: Figure S3 [file peerj-05-3398-s004.pdf]

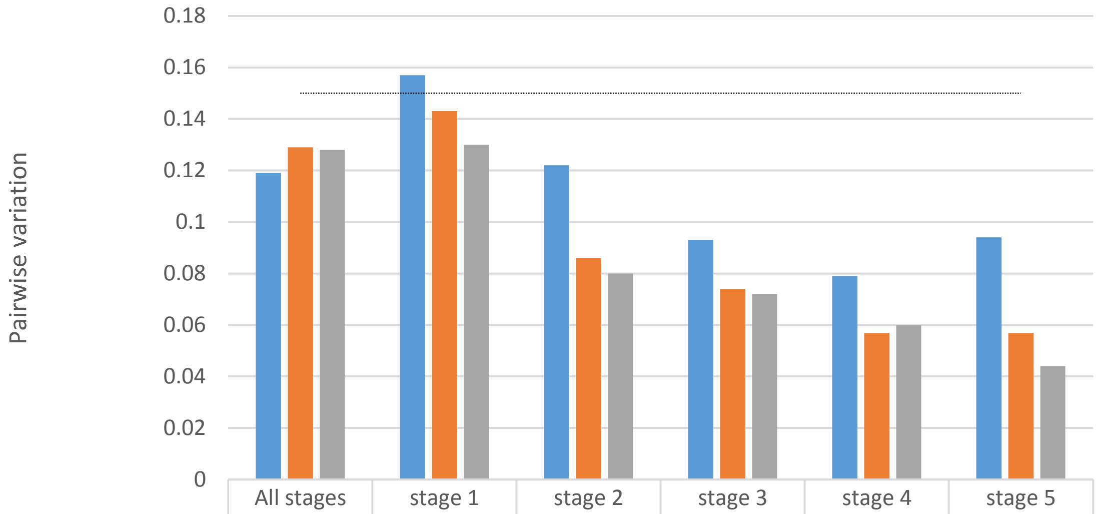

	All stages	stage 1	stage 2	stage 3	stage 4	stage 5
V2/3	0.119	0.157	0.122	0.093	0.079	0.094
V3/4	0.129	0.143	0.086	0.074	0.057	0.057
V4/5	0.128	0.13	0.08	0.072	0.06	0.044
cut-off value	0.15	0.15	0.15	0.15	0.15	0.15

Figure S3. The number of reference genes required for accurate normalization of five developmental stages based on pairwise variation between candidate genes by GeNorm.
