# Supplementary figures and images for: Selection of housekeeping genes as internal controls for quantitative RT-PCR analysis of the veined rapa whelk (Rapana venosa)
Source: PeerJ. 2017 May 31;5:e3398. doi: 10.7717/peerj.3398 (PMC5455708; doi:10.7717/peerj.3398)

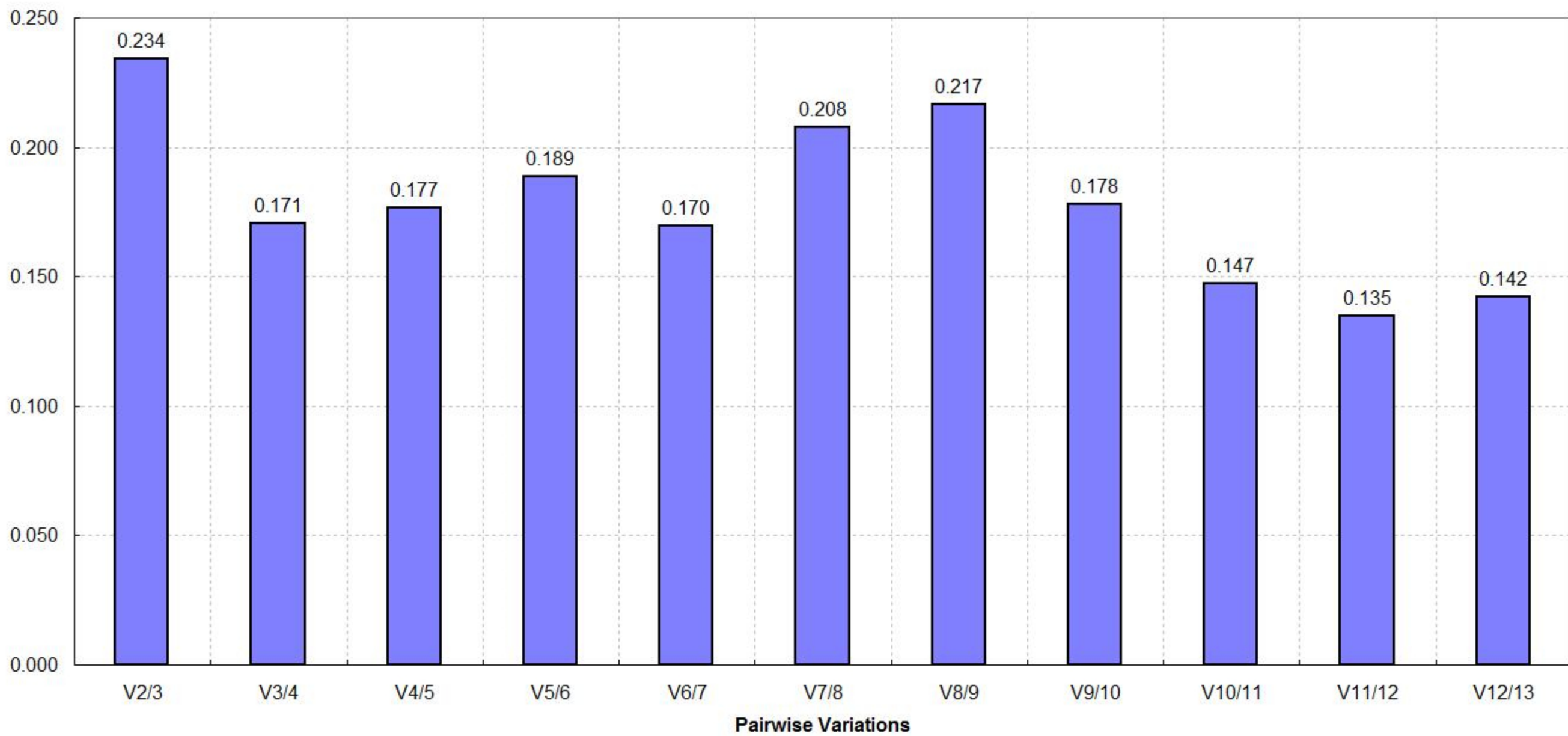

Supplement: Figure S4 [file peerj-05-3398-s005.pdf]
